# False memories for true and false vaccination information form in line with pre‐existing vaccine opinions

**DOI:** 10.1002/acp.4002

**Published:** 2022-10-04

**Authors:** Ciara M. Greene, Constance de Saint Laurent, Karen Hegarty, Gillian Murphy

**Affiliations:** ^1^ School of Psychology University College Dublin Dublin 4 Ireland; ^2^ School of Applied Psychology University College Cork Cork Ireland

**Keywords:** COVID‐19, fake news, false memories, misinformation, vaccine

## Abstract

Misinformation continually threatens efforts to control the COVID‐19 pandemic, with vaccine misinformation now a key concern. False memories for misinformation can influence behavioural intentions, yet little is known about the factors affecting (false) memories for vaccine‐related news items. Across two experiments (total *n* = 1481), this paper explores the effects of pre‐existing vaccine opinions on reported memories for true and false news items. In Study 1, participants (*n* = 817) were exposed to fabricated pro‐ or anti‐vaccine news items, and then asked if they have a memory of this news event having occurred. In Study 2, participants (*n* = 646) viewed true pro‐ or anti‐vaccine news items. News items were more likely to be remembered when they aligned with participants' pre‐existing vaccine beliefs, with stronger effects for pro‐vaccine information. We conclude by encouraging researchers to consider the role of attitudinal bias when developing interventions to reduce susceptibility to misinformation.

## 1 INTRODUCTION

Misleading and biased information has increased in both accessibility and quantity since the introduction of the internet (Lazer et al., 2018). Throughout the COVID‐19 pandemic, online misinformation has proliferated (Brennen et al., 2020; Kouzy et al., 2020), leading the WHO director‐general to declare an ‘infodemic’. As researchers focus on developing the best interventions for the spread and impact of misinformation surrounding COVID‐19, it remains vital to continue to examine the mechanisms underlying why individuals believe, spread, and act on misinformation. Recent research has demonstrated that exposure to ‘fake news’ can result in false memories for the events depicted (Frenda et al., 2013; Greene et al., 2021; Murphy et al., 2019), and that people who form such false memories may be more likely to act on the misinformation (Greene & Murphy, 2021). The unprecedented rise in online COVID‐19 fake vaccine news therefore adds urgency to understanding the factors influencing false memory development.

Recent research indicates that susceptibility to false memories is influenced by a range of individual differences including personality traits, intelligence, and age (Greene et al., 2021; Lee, 2004; Roediger III & Geraci, 2007; Zhu, Chen, Loftus, Lin, He, Chen, et al., 2010; Zhu, Chen, Loftus, Lin, He, Chen, & Dong, 2010). False memories are considerably more likely to form when the news item or event aligns with participants' ideological or political viewpoints (Frenda et al., 2013; Murphy et al., 2019, 2021). Levels of interest in or expertise for a topic have also been linked with higher reporting of false memories for that topic (Baird, 2003; Castel et al., 2007; Mehta et al., 2011; O'Connell & Greene, 2017).

These phenomena may be explained by the source monitoring framework (Johnson et al., 1993; Mitchell & Johnson, 2000), an influential approach which has contributed to current understanding of false memory generation. This framework posits that two cognitive systems operate in parallel to enable individuals to infer the source of mental experiences, and distinguish false from true memories. Individuals evaluate their mental experiences through the first system, relying on heuristic judgements to quickly assess whether the memory's features are characteristic of a true memory; or the second system, relying on systematic and deliberative processes whereby the plausibility of the memory contents are assessed and evaluated against prior knowledge.

Processing by either of these systems can lead to the occurrence of false memories, as individuals incorrectly attribute mental experiences which have been internally generated to actual memories of previous experiences. One example of this which has been extensively studied is inaccuracies in eyewitness testimony; for example, when an eyewitness does not recognise that a mental image of a crime occurring has been prompted by leading questions from the prosecutor, and instead believes it to be a true representation of their own past experience of witnessing the crime (Lindsay, 1994). The individual may then proceed to create a false memory of the crime occurring following this initial inaccurate judgement (Hyman & Kleinknecht, 1999; Mazzoni et al., 2001; Mitchell & Johnson, 2000; Strange et al., 2005).

A similar process may occur when participants are exposed to online misinformation. False memories for a fake news item may occur if a news consumer feels like a fabricated news item is true after initial processing of the story content (i.e. heuristic processing, based on the vividness or clarity of the memory) or may determine that it is likely to be true if the content of the news item corresponds with the news consumer's previous knowledge and experiences (i.e. systematic processing; Mazzoni & Kirsch, 2002). This framework provides an explanation for the finding that false memories tend to form in line with individuals' knowledge, beliefs and interests: prior experiences and knowledge may influence the perceived plausibility of an event, which in turn influences belief that the event actually happened (Mazzoni et al., 2001; Scoboria et al., 2004, 2007).

Fuzzy trace theory (Reyna et al., 2016) provides an alternative theoretical framework, in which false memories arise as a result of parallel formation of ‘verbatim’ and ‘gist’ memory traces for experienced events. While verbatim traces record specific surface details of a given event (e.g. remembering that a pedestrian was struck by a 2018 Toyota Corolla), gist traces provide a boiled‐down representation of the essential characteristics (e.g. remembering a person being hit by a car). According to this model, false memories arise due to overlap in gist representations, such as when a witness confuses their memory of a witnessed traffic accident with another they saw on television; both events contain the same essential features—the gist—but differ in their specific (verbatim) features (Brainerd & Reyna, 2005). Research suggests that people tend to rely on gist representations when making decisions (Reyna & Lloyd, 2006). For example, when faced with the decision to get vaccinated against a disease, an individual will extract the essential features of vaccine‐relevant information they have encountered in the past (i.e. the gist) and use this information in making their decision. Of course, the determination of what features are essential will be determined both by the quality of information available to the individual, and their own background knowledge (Reyna, 2012). Thus, someone who has encountered a lot of anti‐vaccination misinformation and who does not have a strong scientific background may erroneously derive the conclusion that vaccines are unsafe. Upon encountering a new piece of information (e.g. an anti‐vax fake news story) that overlaps substantially with this gist, the individual may falsely remember having encountered the information before.

The tendency to remember items which align with our prior knowledge and experience is not unique to misinformation. Bartlett's (1932) seminal work on schemas demonstrated that memory tends to be stronger for information which aligns with prior knowledge than information that does not. Such findings have been supported by numerous studies (e.g., Anderson, 1981; Tse et al., 2007; van Kesteren et al., 2012). In contrast, evidence that memory may sometimes be enhanced for unexpected or schema‐inconsistent information has also been reported (e.g., Greve et al., 2017; Mäntylä & Bäckman, 1992; Tulving & Kroll, 1995). Greve et al. (2019) explored this apparent contradiction across four experiments with simplified stimuli. They reported a U‐shaped pattern in memory performance, whereby schema‐consistent and schema‐inconsistent information tends to be remembered at higher rates than information that is not relevant to the schema. It remains to be seen whether a similar pattern will be apparent in the reporting of memories for more complex information, such as true and false news items. Moreover, previous work on false memory formation following exposure to ideologically congruent misinformation has focussed on political news. There is as yet no evidence about the effects of pre‐existing opinions on false memories for health‐related information.

### 1.1 The present paper

The COVID‐19 pandemic and rollout of COVID‐19 vaccines is an ideal context for exploring the reporting of memories. Understanding the factors influencing how individuals ‘remember’ both fabricated and accurate news may provide a better understanding of the influence of COVID‐19 vaccine information and misinformation. Based on previous literature, we expect that memories for both true and false information about the COVID‐19 vaccines will be more likely to form in line with participants' pre‐existing views about vaccination. Evidence suggests that the pattern of vaccine hesitancy and anti‐vaccination attitudes may be different for the COVID‐19 vaccine than vaccines in general (Dror et al., 2020; Fridman et al., 2021). We therefore evaluated effects of pre‐existing opinions on vaccination in general, and COVID‐19 vaccination in particular. All measures, manipulations and exclusions are reported. The present studies investigated susceptibility to false memories for fabricated news headlines that were either in favour of or opposed to vaccination against COVID‐19 (Experiment 1) and reporting of memories for accurate COVID‐19 vaccine news headlines (Experiment 2). Only participants who had yet to be vaccinated were included. The hypotheses for these studies are as follows:

- Experiment 1: Participants with more negative attitudes towards vaccines will be more likely to report a memory for fake news stories that are negative about COVID vaccines, while those with more positive attitudes towards vaccines will be more likely to report a memory for fake news stories that are positive about COVID vaccines.
- Experiment 2: Participants with more negative attitudes towards vaccines will be more likely to report a memory for accurate news stories that are negative about COVID vaccines, while those with more positive attitudes towards vaccines will be more likely to report a memory for accurate news stories that are positive about COVID vaccines.

All materials and data for both experiments are available at https://osf.io/jw23x/.

## 2 EXPERIMENT 1

### 2.1 Methods

#### 2.1.1 Pre‐registration

These data were collected as part of a larger study examining effects of misinformation on vaccine intentions (de Saint Laurent et al., 2022). The present paper addressed distinct research questions, and was thus separately preregistered at https://aspredicted.org/55a5y.pdf. Ethical approval was obtained from the Human Research Ethics Committee of University College Dublin.

#### 2.1.2 Participants

Participants (*n* = 1072) were recruited via the platform Prolific (https://www.prolific.co/). Data were collected between 8 June 2021 and 21 June 2021, with participants told the study was focused on media exposure and the COVID‐19 pandemic. Participation was offered to participants in six predominantly English‐speaking countries (Ireland, United Kingdom, New Zealand, Australia, United States, and Canada) who had not received a COVID‐19 vaccine. In line with our preregistration, 220 participants were removed for refusing a post‐debrief consent, failing an attention check, and/or reporting having received at least one dose of a COVID‐19 vaccine.

In order to avoid revealing the true purpose of the experiment, we did not directly ask participants for their views on vaccination, but instead relied on user information collected by Prolific to categorise participants. Participant data on COVID‐19 vaccine opinions was gathered by Prolific, prior to this study, using the question, ‘Please describe your attitudes towards the COVID‐19 (Coronavirus) vaccines’. Response options were ‘I feel positively about the vaccine’ (which we labelled as supports COVID vaccines), ‘I don't have any strong opinions either way’ (labelled as neutral about COVID vaccines), and ‘I feel negatively about the vaccine’ (labelled as against COVID vaccines). Participant data on general vaccine opinions was gathered using the question, ‘Please rate on a scale from 1–7 where 1 is TOTALLY DISAGREE and 7 is TOTALLY AGREE. I believe that scheduled immunizations are safe for children’. For the purposes of the present study, response options 1–3 were labelled anti‐vaccine, option 4 was labelled neutral, and options 5–7 were labelled pro‐vaccine. As some users revoked their consent for this information to be shared with researchers, these participants were removed from all analyses. The final sample (*N* = 817) included 277 (33.9%) males, 534 (65.36%) females and 6 (0.73%) other/prefer to self‐identify. The mean age was 28.52 years (*SD* = 8.91).

#### 2.1.3 Design

This was a between‐subjects design, in which participants were randomly assigned to view either a fake anti‐vaccination headline or fake pro‐vaccination headline, in addition to two true, neutral headlines. A third control condition was included as part of the larger study, but was not relevant to the current study aims and will not be considered here.

#### 2.1.4 Materials

Ten false headlines were fabricated for use in this study, aiming to mimic the type of misinformation that could be found during the period of data collection. To ensure that each headline was indeed false and novel, extensive online searches were conducted to ensure that the events depicted in the fabricated stories had not actually occurred or been reported. Pilot testing was conducted between March and June 2021, and evaluated each headline's plausibility and likelihood to impact someone's decision to get vaccinated. Since the goal of the larger study was to evaluate effects of misinformation on vaccination intentions, the headlines most likely to affect behaviour were chosen for use in the main study. Specifically, we selected the pro‐vaccine headlines which scored the highest, and the anti‐vaccine headlines which scored the lowest for behavioural inclination. The headlines were balanced across conditions for plausibility and likelihood to impact behavioural intention to get vaccinated. Importantly, the fabricated headlines all focussed on scientific information and details of the vaccine development, and were not explicitly political in nature.^1^ Five pro‐vaccine headlines (e.g. ‘New study finds risk of lung cancer to be significantly reduced after two shots of COVID‐19 vaccine’) and five anti‐vaccine headlines (e.g. ‘The mRNA technology in the COVID‐19 vaccine affects cell mutation and decreases your bone density’) were selected; see supplementary materials, Data S1 for a complete list of all headlines used. Each fabricated headline was accompanied by a stock image of a vaccine vial.

Five true, neutral headlines were also created, describing actual events relating to the COVID‐19 pandemic that were deemed unlikely to influence vaccination decisions (e.g. ‘Production for the new Batman movie to be released in 2022 was halted when its star, Robert Pattinson, tested positive for COVID‐19’). Similar to the fabricated stories, each headline was accompanied by a relevant image (e.g. a picture of Robert Pattinson dressed as Batman).

#### 2.1.5 Procedure

The study was presented to participants as aiming ‘to investigate reactions to a range of news stories relating to the novel coronavirus outbreak’, with neither fake news nor misinformation being mentioned. After consenting to the study, participants reported demographic information (age and gender), and whether they had received at least one dose of the COVID‐19 vaccine. Only unvaccinated participants were eligible to continue. They were then randomly assigned to the anti‐vaccine or pro‐vaccine condition. Participants then viewed three news headlines, in random order. These included one fake headline, randomly selected from the appropriate list of pro‐ or anti‐vaccination headlines, and two randomly selected neutral headlines. Each headline was presented on a separate page, illustrated by an image, and accompanied by the question ‘Do you remember the events described in this story?’. The four possible responses were: ‘I have a clear memory of seeing/hearing about this’, ‘I have a vague memory of this happening’, ‘I remember this differently’, and ‘I don't remember this’. As part of a larger study, intentions to engage in a range of health behaviours were examined, including intentions to receive the COVID‐19 vaccine. These data are reported elsewhere (de Saint Laurent et al., 2022).

Participants were then debriefed and were re‐presented with the headlines accompanied with an explanation of whether each headline was fabricated or true. Following the full debrief, during which participants were informed of the true nature of the study, participants were asked to consent once more to the use of their data for analysis.

## 3 RESULTS

Based on data collected by Prolific, 381 (46.63%) of participants reported being in favour of COVID‐19 vaccines, with 288 (35.25%) neutral about them and 148 participants (18.12%) against them. With respect to general vaccines, 623 (76.25%) of participants were categorised as pro‐vaccine, with 91 (11.14%) identified as anti‐vaccine and 103 (12.61%) neutral on the subject.

### 3.1 False memories for novel misinformation

Answers to the questions ‘Do you remember the events described in this story?’ were recoded as a binary variable. In line with our preregistration, the responses ‘I have a clear memory of seeing/hearing about this’ or ‘I have a vague memory of this happening’ were coded as remembering the headline, and ‘I remember this differently’ or ‘I don't remember this’ were coded as not remembering it. Of the participants exposed to novel misinformation about COVID‐19 vaccines, 9.7% reported remembering the anti‐vaccine headlines while 15.0% remembered the pro‐vaccine headlines. In comparison, the accurate headlines were remembered, on average, by 51.9% of participants.

**FIGURE 1:**
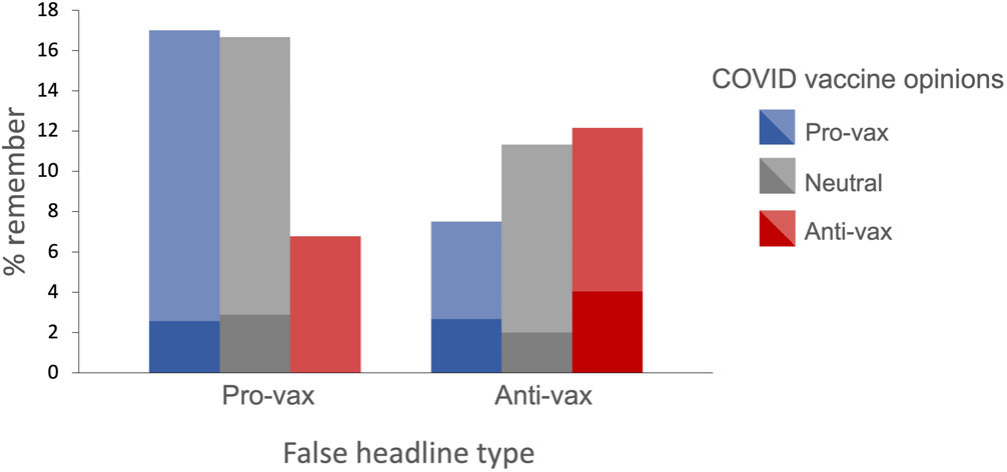
Percentage of participants reporting false memories for fabricated pro‐ and anti‐vax headlines, split by pre‐existing COVID‐19 vaccine opinions. The relative frequency of specific memories (dark colours, bottom) and vague memories (light colours, top) may also be seen.

Table 1 provides a breakdown of false memory rates across general and COVID‐19 vaccine opinion categories. For both conditions, participants were more likely to falsely remember headlines aligned with their pre‐existing COVID‐19 and general vaccine opinions (see Figure 1; in the interests of clarity, the data in this figure are collapsed across general vaccine opinion categories).

**TABLE 1:** Percentage of participants who reported remembering the fabricated pro‐ and anti‐vaccine headlines, categorised by general vaccine and COVID‐19 vaccine opinions

	COVID‐19 vaccine opinions							
	Supports COVID‐19 vaccines		Neutral about COVID‐19 vaccine		Against COVID‐19 vaccines		*All COVID‐19 vaccine opinions*	
General vaccine opinions	Total *n*	False memory *n* (%)	Total *n*	False memory *n* (%)	Total *n*	False memory *n* (%)	*Total n*	*False memory n (%)*
Pro‐vaccine false headlines								
Pro‐vaccine opinions	184	30 (16.3%)	93	14 (14.9%)	29	3 (10.3%)	307	47 (15.3%)
Neutral vaccine opinions	6	1 (16.7%)	36	6 (16.7%)	11	1 (9.1%)	53	8 (15.1%)
Anti‐vaccine opinions	4	2 (50%)	8	3 (37.5%)	34	1 (2.9%)	46	6 (13.0%)
All general vaccine opinions	194	33 (17%)	138	23 (16.7%)	74	5 (6.8%)	406	61 (15.0%)
Anti‐vaccine false headlines								
Pro‐vaccine opinions	176	12 (6.8%)	101	12 (11.9%)	39	4 (10.3%)	316	28 (8.9%)
Neutral vaccine opinions	9	1 (11.1%)	33	4 (12.1%)	8	0 (0.0%)	50	5 (10.0%)
Anti‐vaccine opinions	2	1 (50.0%)	16	1 (6.3%)	27	5 (18.5%)	45	7 (15.6%)
All general vaccine opinions	187	14 (7.5%)	150	17 (11.3%)	74	9 (12.2%)	411	40 (9.7%)

In order to evaluate the effect of pre‐existing vaccine opinions on false memories of novel headlines, we conducted two binary logistic regressions, one for the anti‐vaccine headlines and one for the pro‐vaccine headlines. Each used the participants' pre‐existing opinions about general vaccines and COVID‐19 vaccines as the predictor variables, and whether they reported remembering seeing the novel headlines as the outcome variable. The overall models were not statistically significant (anti‐vaccine: *χ* ^2^(2, *N* = 411) = 2.00, *p* = .37, Cox‐Snell *R* ^2^ < .01, Nagelkerke *R* ^2^ = .01; pro‐vaccine: *χ* ^2^(2, *N* = 406) = 4.9, *p* = .085, Cox‐Snell *R* ^2^ = .01, Nagelkerke *R* ^2^ = .02). Individual coefficients are listed in Table 2. The only significant predictor was COVID‐19 vaccine opinions for the pro‐vaccine headlines (*p* = .027), such that participants with positive pre‐existing views of COVID‐19 vaccination were more likely to falsely remember the fabricated pro‐vaccination headline.

**TABLE 2:** Results of a logistic regression evaluating effects of pre‐existing vaccine opinions on false memories of fabricated pro‐ and anti‐vaccine headlines

Predictor	*b*	SE *b*	*df*	Wald	*p*‐value	OR	95% CI
Pro‐vaccine false headlines							
Intercept	−1.12	0.57	1	3.8	.051	0.33	
COVID‐19 vaccine opinions	0.56	0.25	1	4.9	.027	1.75	[1.08, 2.91]
General vaccine opinions	−0.15	0.11	1	1.8	.183	0.86	[0.69, 1.08]
Anti‐vaccine false headlines							
Intercept	−1.86	0.64	1	8.6	.003	0.16	
COVID‐19 vaccine opinions	−0.22	0.26	1	0.68	.410	0.81	[0.49, 1.36]
General vaccine opinions	−0.06	0.12	1	0.25	.620	0.94	[0.75, 1.20]

The sample in the present analysis was somewhat unbalanced, with the majority of participants reporting pro‐vaccination opinions. In order to increase statistical power, we therefore conducted an additional analysis, not included in the preregistration. The congruence between the headline and the participants' pre‐existing COVID‐19 vaccine opinion was calculated. Only participants who reported pro‐ or anti‐vaccine attitudes were included, excluding those who reported neutral views. A chi square analysis was then carried out, comparing the frequency of false memories for attitudinally congruent and incongruent headlines. A significant difference between groups was observed, such that participants were more likely to report false memories for attitudinally congruent headlines (*χ* ^2^ (1, 529) = 9.13, *p* = .003, Cramer's *V* = 0.13). Congruent headlines were remembered by 15.7% of participants, compared to 7.3% of participants remembering headlines incongruent with their COVID‐vaccine attitudes (see Figure 1).

## 4 EXPERIMENT 1 DISCUSSION

The results of Experiment 1 demonstrate that individuals are more likely to report remembering a fabricated COVID‐19 vaccine headline if the content of the headline corresponds with their pre‐existing opinion on COVID‐19 vaccines. This effect was only observed for pro‐vaccination headlines in the preregistered logistic regression—most likely due to the small number of participants who identified as having anti‐vaccination beliefs—however a chi square analysis which collapsed across opinion categories demonstrated a clear effect of attitudinal congruency.

Such results support the source monitoring framework, which posits that pre‐existing attitudes and opinions can influence the formation of false memories. The findings can also be explained via fuzzy trace theory, if the gist of the fake news story overlaps with other related memories. These results are in line with previous studies which have linked attitudinal congruence with the formation of false memories for fake news about an abortion referendum (Murphy et al., 2019), the feminist movement (Murphy et al., 2021) and US politics (Frenda et al., 2013). Experiment 1 dealt exclusively with fabricated stories about events that never took place. The source monitoring framework predicts that memory reconstruction is prone to systematic bias, and that this should be the case regardless of the ground truth of the item under consideration. Thus, in Experiment 2, we hypothesise that a similar pattern will be observed for reported memories of true pro‐ and anti‐vaccination headlines.

## 5 EXPERIMENT 2

### 5.1 Methods

#### 5.1.1 Participants

Data were collected from 792 participants between 18 June 2021 and 19 June 2021. Participant recruitment and selection filters (location and vaccine status) were the same as Experiment 1, with no participants having taken part in Experiment 1. As in Experiment 1, participants' pre‐existing general vaccine and COVID‐19 vaccine opinions were collected through data made available by Prolific. In Experiment 1, we observed that anti‐vaccination attitudes were relatively infrequent, with consequences for our planned statistical analyses. In Experiment 2, we therefore applied additional filters via Prolific to ensure a well‐balanced sample for COVID‐19 vaccine attitudes. Following the eligibility criteria for Experiment 1, 146 participants were removed (134 had received at least one vaccine dose, 15 failed the attention check). The final sample included 646 participants (402 females, 232 males, 12 other; M age = 30.49, *SD* = 10.17).

#### 5.1.2 Design

This study employed a between‐subjects design, where participants were randomly assigned to view either a true anti‐vaccination headline or a true pro‐vaccination headline, in addition to two neutral true stories about COVID‐19.

Ethical approval was obtained from the Human Research Ethics Committee University College Dublin.

#### 5.1.3 Materials

Ten true pro‐ and anti‐vaccination headlines were compiled for use in this study. The information contained in the headlines was obtained from reliable news sources and based on accurate information, although the specific phrasing was amended for the purpose of this study. Similar to Experiment 1, the headlines were piloted between March and June 2021 for plausibility and likelihood to impact someone's decision to get vaccinated, and both plausibility and behavioural impact were balanced across conditions. Five anti‐vaccine headlines (e.g. ‘COVID vaccines associated with false‐positive breast cancer result’) and five pro‐vaccine headlines (e.g. ‘Vaccines may provide coronavirus immunity that lasts for years, finds study’) were selected for use in the main study. Each headline was accompanied by stock image of a vaccine vial (see supplementary materials for all headlines).

The five neutral true stories employed in Experiment 1 were re‐used in this experiment.

#### 5.1.4 Procedure

The procedure was identical to Experiment 1, with the exception of the headlines used.

## 6 RESULTS

Preliminary analysis revealed that 215 (33.3%) participants were against, 215 (33.3%) were neutral and 216 (33.4%) were in favour of COVID‐19 vaccination. 443 (68.58%) of participants were pro‐vaccine in general, with 98 (15.17%) neutral about them and 105 (16.25%) anti‐vaccine.

### 6.1 Memories for headlines

**FIGURE 2:**
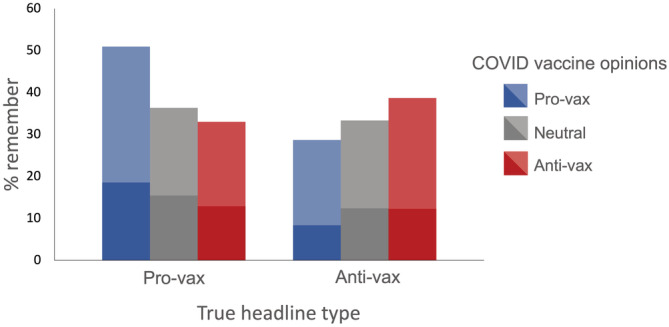
Percentage of participants reporting memories for true pro‐ and anti‐vaccination headlines, split by pre‐existing opinions regarding COVID‐19 vaccination. The relative frequency of specific memories (dark colours, bottom) and vague memories (light colours, top) may also be seen.

Responses were coded as ‘remembered’/‘did not remember’ in the same manner as in Experiment 1. Overall, 33.5% of participants reported remembering the anti‐vaccine headlines, while 40.1% remembered the pro‐vaccine headlines. The neutral headlines were remembered, on average, by 51.9% of the participants. For both conditions, participants were more likely to remember headlines that aligned with their pre‐existing COVID‐19 and general vaccine opinions (see Figure 2). Table 3 provides a breakdown of the rates of reported memories across general and COVID‐19 vaccine opinion categories.

**TABLE 3:** Number and percentage of true pro‐ and anti‐vaccine headlines remembered, categorised by general vaccine and COVID vaccine opinions

	COVID‐19 vaccine opinions							
	Supports COVID‐19 vaccines		Neutral about COVID‐19 vaccine		Against COVID‐19 vaccines		*All COVID‐19 vaccine opinions*	
General vaccine opinions	*n*	Memory *n* (%)	*n*	Memory *n* (%)	*n*	% rem.	*n*	*% rem*.
Pro‐vaccination true headlines								
Pro‐vaccine opinions	99	50 (50.5%)	83	31 (37.3%)	47	14 (29.8%)	229	95 (41.5%)
Neutral vaccine opinions	6	3 (50.0%)	20	7 (35.0%)	19	5 (26.3%)	45	15 (33.3%)
Anti‐vaccine opinions	3	2 (66.7%)	7	2 (28.6%)	43	17 (39.5%)	53	21 (39.6%)
All general vaccine opinions	108	55 (50.9%)	110	40 (36.4%)	109	36 (33.0%)	327	131 (40.1%)
Anti‐vaccination true headlines								
Pro‐vaccine opinions	101	27 (26.7%)	70	29 (41.4%)	43	19 (44.2%)	214	75 (35.0%)
Neutral vaccine opinions	6	3 (50.0%)	28	6 (21.4%)	19	4 (21.1%)	53	13 (24.5%)
Anti‐vaccine opinions	1	1 (100%)	7	0 (0.0%)	44	18 (40.9%)	52	18 (35.0%)
All general vaccine opinions	108	31 (28.7%)	105	35 (33.3%)	106	41 (38.68%)	319	107 (33.5%)

Binary logistic regressions evaluated the impact of pre‐existing vaccine opinions on memories for accurate headlines, separately for pro‐ and anti‐vax stories. The overall model, including COVID‐19 vaccine opinions and general vaccine opinions, was significant for pro‐vaccine headlines (*χ* ^2^[2, *N* = 327] = 7.5, *p* = .024, Cox‐Snell *R* ^2^ = .02, Nagelkerke *R* ^2^ = .03), but not for anti‐vax headlines (*χ* ^2^[2, *N* = 319] = 3.1, *p* = .210, Cox‐Snell *R* ^2^ = .01, Nagelkerke *R* ^2^ = .01). Individual coefficients are presented in Table 4. As in Experiment 1, COVID‐19 vaccine opinions significantly predicted memories for pro‐vaccine headlines (*p* = .01) but not for anti‐vaccine headlines (*p* = .08).

**TABLE 4:** Results of a logistic regression evaluating effects of pre‐existing vaccine opinions on memories of true pro‐ and anti‐vaccine headlines

Predictor	*b*	SE *b*	*df*	Wald	*p*‐value	OR	95% CI
Pro‐vaccination true headlines							
Intercept	−0.17	0.41	1	0.16	.690	0.85	
COVID‐19 vaccine opinions	0.43	0.17	1	6.60	.010	1.54	[1.11, 2.15]
General vaccine opinions	−0.04	0.08	1	0.38	.540	0.95	[0.82, 1.10]
Anti‐vaccination true headlines							
Intercept	−1.06	0.44	1	5.7	.017	0.35	
COVID‐19 vaccine opinions	−0.32	0.18	1	3.1	.078	0.73	[0.51, 1.04]
General vaccine opinions	0.07	0.08	1	0.76	.380	1.07	[0.91, 1.27]

As with Experiment 1, a follow‐up analysis was conducted to maximise power, in which participants were grouped based on whether they had viewed a headline that was congruent or incongruent with their pre‐existing opinion on COVID‐19 vaccines. Participants who reported feeling neutral on the issue of COVID‐19 vaccines were excluded from this analysis. A significant effect of congruency was observed: memories were reported for 44.86% of congruent headlines, compared with just 30.88% of incongruent headlines (*χ* ^2^ [1, 431] = 8.96, *p* = .003, Cramer's *V* = 0.14).

## 7 GENERAL DISCUSSION

The aim of this study was to investigate the effect of pre‐existing vaccine attitudes on reporting of memories for fabricated and actual COVID‐19 vaccine news items. Our findings suggest that individuals are more likely to report a memory for both accurate and fabricated vaccine news items which align with their pre‐existing (i.e. schema‐consistent) opinions about COVID‐19 vaccination. The effect was specific to attitudes towards COVID‐19 vaccination; general vaccine attitudes had little effect on the probability of reporting a memory for both accurate and fabricated news items.

Importantly, the overall effect of attitudinal congruence was observed in an exploratory analysis collapsing across misinformation type. In the preregistered analyses, a significant effect of misinformation was observed only for pro‐vaccine headlines. In the case of Experiment 1 (evaluating the effect of fake news headlines), this may be due to the relative infrequency of participants reporting anti‐vaccination views. A similar pattern of results was observed in Experiment 2 (examining responses to true news headlines). While a significant effect of attitudinal congruency was observed overall, this effect only reached significance for pro‐vaccine information. This finding was somewhat unexpected: we predicted a symmetrical effect for both pro‐ and anti‐vaccination information. The expected pattern of results was observed, but the effect was smaller for anti‐vaccine information across both experiments. Given the significant efforts by governments and health agencies worldwide to suppress COVID misinformation and disseminate accurate information about the pandemic (e.g. WHO, 2021), participants are likely to have encountered considerably more pro‐vaccine information in their daily media diets (see Cinelli et al., 2020 for an analysis of the spread of COVID misinformation on social media). As a result participants are likely to have been more familiar with the general tenor of pro‐vaccination arguments. It is also possible that they were simply more convinced by pro‐vaccine information, regardless of their own position on the issue, and therefore more likely to (falsely) recall having seen it before.

The current research adds to literature suggesting that congruence of a news item with pre‐existing beliefs may be a significant contributing factor to false memory formation (Frenda et al., 2013; Greene et al., 2021; Murphy et al., 2019, 2021). This paper builds on recent research which has examined the influence of congruency on simple item‐pairing paradigms (e.g. Greve et al., 2019), and provides the first specific evidence of this effect in the context of non‐partisan health misinformation. Such findings may be explained via the source monitoring framework; if prior knowledge and opinions guide judgements surrounding memories for past events (e.g. plausibility), source monitoring failures may be more likely to occur for information which is attitudinally congruent (Johnson et al., 1993). The finding may also be explained in terms of fuzzy‐trace theory, whereby specific, verbatim features of the news stories are disregarded in favour of the underlying gist (i.e. ‘COVID vaccines are good’; ‘COVID vaccines are bad’). As the news items in Experiment 2 depicted actual COVID‐19 vaccine news events, it appears that attitudinal congruence and corresponding overlap in gist representations may be a significant contributing factor in ‘remembering’ news items regardless of their basis in truth.

Participants were more likely to report a memory for the true news items presented in Experiment 2 than for the fabricated items in Experiment 1. This may simply be due to the fact that participants had actually encountered these items in the past and were correctly reporting their memories. Given the rate of false memory reports observed in Experiment 1 however, it is likely that at least some of the memory reports for real events in Experiment 2 represent false memories. The current study used a relatively simple paradigm, following similar false‐memory studies (e.g. Frenda et al., 2013; Greene & Murphy, 2020; Murphy et al., 2021). The richness of the reported memories for both the accurate and fabricated news items was not assessed, and we cannot comment on whether memories for true and false events were qualitatively distinct. Moreover, we cannot rule out the possibility that some the memory reports in the present study actually reflect participant beliefs that the event occurred, rather than memories of encountering the event in the past (c.f. Wade et al., 2018). Nevertheless, it is clear that participants' willingness to endorse a statement of memory for news events was influenced by their existing beliefs about vaccines, regardless of the truth of the news items.

Multiple exposures to inaccurate information can increase the perceived truthfulness of the information (Fazio et al., 2019; Pennycook et al., 2018), and may have consequences for memory formation too. In order to control for this ‘illusory truth effect’, novel fake news stories were created for Experiment 1. As all of the events described in Experiment 2 were real news events, it was not possible to control for the influence of previous multiple exposures to the content of the news headlines. It is therefore recommended that future research examine the influence of pre‐existing vaccine attitudes on memory reports for vaccine‐related news items using paradigms which can control for the influence of previous exposure and which allow for deeper exploration of these memory reports (e.g. longitudinal designs assessing memories over time).

As the current study indicates that individuals are more susceptible to reporting memories or beliefs for both accurate and fabricated vaccine news items which align with their pre‐existing vaccine attitudes, the role of attitudinal bias should be considered when developing interventions focused on news literacy and reducing susceptibility to misinformation.

## FUNDING INFORMATION

This research was funded by a grant from the Health Research Board of Ireland to CG and GM, grant code COV19‐2020‐030. The funders had no role in the study design, collection, analysis and interpretation of data, writing of the report or decision to submit the article for publication.

## CONFLICT OF INTEREST

The authors declare no conflict of interest.

## Supporting information


**Appendix S1:** Supporting Information.

## Data Availability

All materials and data for both experiments are available at https://osf.io/jw23x/.

## Endnote

^1^
Beliefs about vaccine and vaccine hesitancy tend to be strongly correlated with political orientation in the United States (Albrecht, 2022), and might therefore be considered somewhat political in nature, however this relationship is much weaker in other English‐speaking countries such as the United Kingdom and Canada (Pennycook et al., 2022), from which the sample was mostly drawn.

## References

[acp4002-bib-0001] Albrecht, D. (2022). Vaccination, politics and COVID‐19 impacts. BMC Public Health, 22(1), 96. 10.1186/s12889-021-12432-x 35031053 PMC8758893

[acp4002-bib-0002] Anderson, J. R. (1981). Effects of prior knowledge on memory for new information. Memory & Cognition, 9(3), 237–246.

[acp4002-bib-0003] Baird, R. R. (2003). Experts sometimes show more false recall than novices: A cost of knowing too much. Learning and Individual Differences, 13(4), 349–355.

[acp4002-bib-0004] Bartlett, F. C. (1932). Remembering: A study in experimental and social psychology. Cambridge University Press.

[acp4002-bib-0005] Brainerd, C. J. , & Reyna, V. F. (2005). The science of false memory. Oxford University Press.

[acp4002-bib-0006] Brennen, J. S. , Simon, F. M. , Howard, P. N. , & Nielsen, R. K. (2020). Types, sources, and claims of COVID‐19 misinformation Doctoral dissertation. University of Oxford.

[acp4002-bib-0007] Castel, A. D. , McCabe, D. P. , Roediger, H. L., III , & Heitman, J. L. (2007). The dark side of expertise: Domain‐specific memory errors. Psychological Science, 18(1), 3–5. 17362368 10.1111/j.1467-9280.2007.01838.x

[acp4002-bib-0008] Cinelli, M. , Quattrociocchi, W. , Galeazzi, A. , Valensise, C. M. , Brugnoli, E. , Schmidt, A. L. , Zola, P. , Zollo, F. , & Scala, A. (2020). The COVID‐19 social media infodemic. Scientific Reports, 10(1), 1–10. 33024152 10.1038/s41598-020-73510-5 PMC7538912

[acp4002-bib-0009] de Saint Laurent, C. , Murphy, G. , Hegarty, K. , & Greene, C. M. (2022). Measuring the effects of misinformation exposure and beliefs on behavioural intentions: A COVID‐19 vaccination study. Cognitive Research: Principles and Implications *7*, 87. 10.1186/s41235-022-00437-y PMC9526535 36183027

[acp4002-bib-0010] Dror, A. A. , Eisenbach, N. , Taiber, S. , Morozov, N. G. , Mizrachi, M. , Zigron, A. , Srouji, S. , & Sela, E. (2020). Vaccine hesitancy: The next challenge in the fight against COVID‐19. European Journal of Epidemiology, 35(8), 775–779. 32785815 10.1007/s10654-020-00671-y PMC8851308

[acp4002-bib-0011] Fazio, L. K. , Rand, D. G. , & Pennycook, G. (2019). Repetition increases perceived truth equally for plausible and implausible statements. Psychonomic Bulletin & Review, 26(5), 1705–1710. 31420808 10.3758/s13423-019-01651-4

[acp4002-bib-0012] Frenda, S. J. , Knowles, E. D. , Saletan, W. , & Loftus, E. F. (2013). False memories of fabricated political events. Journal of Experimental Social Psychology, 49(2), 280–286.

[acp4002-bib-0013] Fridman, A. , Gershon, R. , & Gneezy, A. (2021). COVID‐19 and vaccine hesitancy: A longitudinal study. PLoS One, 16(4), e0250123. 33861765 10.1371/journal.pone.0250123 PMC8051771

[acp4002-bib-0014] Greene, C. M. , & Murphy, G. (2020). Individual differences in susceptibility to false memories for COVID‐19 fake news. Cognitive Research: Principles and Implications, 5(1), 1–8. 33275199 10.1186/s41235-020-00262-1 PMC7716111

[acp4002-bib-0048] Greene, C. M., & Murphy, G. (2021). Quantifying the effects of fake news on behavior: Evidence from a study of COVID‐19 misinformation. Journal of Experimental Psychology: Applied, 27(4), 773–784. 10.1037/xap0000371 34110860

[acp4002-bib-0015] Greene, C. M. , Nash, R. A. , & Murphy, G. (2021). Misremembering Brexit: Partisan bias and individual predictors of false memories for fake news stories among Brexit voters. Memory, 29, 1–18. 33971789 10.1080/09658211.2021.1923754

[acp4002-bib-0016] Greve, A. , Cooper, E. , Kaula, A. , Anderson, M. C. , & Henson, R. (2017). Does prediction error drive one‐shot declarative learning? Journal of Memory and Language, 94, 149–165. 28579691 10.1016/j.jml.2016.11.001 PMC5381756

[acp4002-bib-0017] Greve, A. , Cooper, E. , Tibon, R. , & Henson, R. N. (2019). Knowledge is power: Prior knowledge aids memory for both congruent and incongruent events, but in different ways. Journal of Experimental Psychology: General, 148(2), 325–341. 30394766 10.1037/xge0000498 PMC6390882

[acp4002-bib-0018] Hyman, I. E. , & Kleinknecht, E. E. (1999). False childhood memories: Research, theory, and applications. In L. M. Williams & V. L. Banyard (Eds.), Trauma and memory (pp. 175–188). Sage.

[acp4002-bib-0019] Johnson, M. K. , Hashtroudi, S. , & Lindsay, D. S. (1993). Source monitoring. Psychological Bulletin, 114(1), 3–28. 8346328 10.1037/0033-2909.114.1.3

[acp4002-bib-0020] Kouzy, R. , Abi Jaoude, J. , Kraitem, A. , El Alam, M. B. , Karam, B. , Adib, E. , et al. (2020). Coronavirus goes viral: Quantifying the COVID‐19 misinformation epidemic on twitter. Cureus, 12, 3. 10.7759/cureus.7255 PMC7152572 32292669

[acp4002-bib-0021] Lazer, D. M. , Baum, M. A. , Benkler, Y. , Berinsky, A. J. , Greenhill, K. M. , Menczer, F. , Metzger, M. J. , Nyhan, B. , Pennycook, G. , & Rothschild, D. (2018). The science of fake news. Science, 359(6380), 1094–1096. 29590025 10.1126/science.aao2998

[acp4002-bib-0022] Lee, K. (2004). Age, neuropsychological, and social cognitive measures as predictors of individual differences in susceptibility to the misinformation effect. Applied Cognitive Psychology, 18(8), 997–1019.

[acp4002-bib-0023] Lindsay, D. S. (1994). Memory source monitoring and eyewitness testimony. In D. F. In , J. D. R. Ross , & M. P. Toglia (Eds.), Adult eyewitness testimony: Current trends and developments (pp. 27–55). Cambridge University Press.

[acp4002-bib-0026] Mazzoni, G. A. , Loftus, E. F. , & Kirsch, I. (2001). Changing beliefs about implausible autobiographical events: A little plausibility goes a long way. Journal of Experimental Psychology: Applied, 7(1), 51. 11577619

[acp4002-bib-0025] Mazzoni, G. , & Kirsch, I. (2002). Autobiographical memories and beliefs: A preliminary metacognitive model. In T. J. Perfect & B. L. Schwartz (Eds.), Applied metacognition (pp. 121–145). Cambridge University Press.

[acp4002-bib-0027] Mehta, R. , Hoegg, J. , & Chakravarti, A. (2011). Knowing too much: Expertise‐induced false recall effects in product comparison. Journal of Consumer Research, 38(3), 535–554.

[acp4002-bib-0028] Mitchell, K. J. , & Johnson, M. K. (2000). Source monitoring: Attributing mental experiences. In E. Tulving & F. I. M. Craik (Eds.), The oxford handbook of memory (pp. 179–195). Oxford University Press.

[acp4002-bib-0029] Murphy, G. , Loftus, E. F. , Grady, R. H. , Levine, L. J. , & Greene, C. M. (2019). False memories for fake news during Ireland's abortion referendum. Psychological Science, 30(10), 1449–1459. 31432746 10.1177/0956797619864887

[acp4002-bib-0030] Murphy, G. , Murray, E. , & Gough, D. (2021). Attitudes towards feminism predict susceptibility to feminism‐related fake news. Applied Cognitive Psychology, 35, 1182–1192.

[acp4002-bib-0024] Mäntylä, T. , & Bäckman, L. (1992). Aging and memory for expected and unexpected objects in real‐world settings. Journal of Experimental Psychology: Learning, Memory, and Cognition, 18(6), 1298–1309. 1447553 10.1037//0278-7393.18.6.1298

[acp4002-bib-0031] O'Connell, A. , & Greene, C. M. (2017). Not strange but not true: Self‐reported interest in a topic increases false memory. Memory, 25(8), 969–977. 27710198 10.1080/09658211.2016.1237655

[acp4002-bib-0032] Pennycook, G. , Cannon, T. D. , & Rand, D. G. (2018). Prior exposure increases perceived accuracy of fake news. Journal of Experimental Psychology: General, 147(12), 1865–1880. 30247057 10.1037/xge0000465 PMC6279465

[acp4002-bib-0033] Pennycook, G. , McPhetres, J. , Bago, B. , & Rand, D. G. (2022). Beliefs about COVID‐19 in Canada, the United Kingdom, and the United States: A novel test of political polarization and motivated reasoning. Personality and Social Psychology Bulletin, 48(5), 750–765. 10.1177/01461672211023652 34180276 PMC9066691

[acp4002-bib-0034] Reyna, V. F. (2012). Risk perception and communication in vaccination decisions: A fuzzy‐trace theory approach. Vaccine, 30(25), 3790–3797. 22133507 10.1016/j.vaccine.2011.11.070 PMC3330177

[acp4002-bib-0036] Reyna, V. F. , & Lloyd, F. J. (2006). Physician decision making and cardiac risk: Effects of knowledge, risk perception, risk tolerance, and fuzzy processing. Journal of Experimental Psychology: Applied, 12(3), 179. 16953744 10.1037/1076-898X.12.3.179

[acp4002-bib-0035] Reyna, V. F. , Corbin, J. C. , Weldon, R. B. , & Brainerd, C. J. (2016). How fuzzy‐trace theory predicts true and false memories for words, sentences, and narratives. Journal of Applied Research in Memory and Cognition, 5(1), 1–9. 27042402 10.1016/j.jarmac.2015.12.003 PMC4815269

[acp4002-bib-0037] Roediger, H. L., III , & Geraci, L. (2007). Aging and the misinformation effect: A neuropsychological analysis. Journal of Experimental Psychology: Learning, Memory, and Cognition, 33(2), 321. 17352614 10.1037/0278-7393.33.2.321

[acp4002-bib-0038] Scoboria, A. , Lynn, S. J. , Hessen, J. , & Fisico, S. (2007). So that's why I don't remember: Normalising forgetting of childhood events influences false autobiographical beliefs but not memories. Memory, 15(8), 801–813. 17943606 10.1080/09658210701685266

[acp4002-bib-0039] Scoboria, A. , Mazzoni, G. , Kirsch, I. , & Relyea, M. (2004). Plausibility and belief in autobiographical memory. Applied Cognitive Psychology, 18(7), 791–807.

[acp4002-bib-0040] Strange, D. , Gerrie, M. P. , & Garry, M. (2005). A few seemingly harmless routes to a false memory. Cognitive Processing, 6(4), 237–242. 18239952 10.1007/s10339-005-0009-7

[acp4002-bib-0041] Tse, D. , Langston, R. F. , Kakeyama, M. , Bethus, I. , Spooner, P. A. , Wood, E. R. , Witter, M. P. , & Morris, R. G. (2007). Schemas and memory consolidation. Science, 316(5821), 76–82. 17412951 10.1126/science.1135935

[acp4002-bib-0042] Tulving, E. , & Kroll, N. (1995). Novelty assessment in the brain and long‐term memory encoding. Psychonomic Bulletin & Review, 2(3), 387–390. 24203720 10.3758/BF03210977

[acp4002-bib-0043] Van Kesteren, M. T. , Ruiter, D. J. , Fernández, G. , & Henson, R. N. (2012). How schema and novelty augment memory formation. Trends in Neurosciences, 35(4), 211–219. 22398180 10.1016/j.tins.2012.02.001

[acp4002-bib-0044] Wade, K. A. , Garry, M. , & Pezdek, K. (2018). Deconstructing rich false memories of committing crime: Commentary on Shaw and Porter (2015). Psychological Science, 29(3), 471–476. 29315022 10.1177/0956797617703667

[acp4002-bib-0045] World Health Organisation (2021, April 27). Fighting misinformation in the time of COVID‐19, one click at a time. https://www.who.int/news-room/feature-stories/detail/fighting-misinformation-in-the-time-of-covid-19-one-click-at-a-time.

[acp4002-bib-0047] Zhu, B. , Chen, C. , Loftus, E. F. , Lin, C. , He, Q. , Chen, C. , He, L. , Moyzisc, R. K. , Lessard, J. , & Dong, Q. (2010). Individual differences in false memory from misinformation: Personality characteristics and their interactions with cognitive abilities. Personality and Individual Differences, 48(8), 889–894.

[acp4002-bib-0046] Zhu, B. , Chen, C. , Loftus, E. F. , Lin, C. , He, Q. , Chen, C. , Li, H. , Xue, G. , Lu, Z. , & Dong, Q. (2010). Individual differences in false memory from misinformation: Cognitive factors. Memory, 18(5), 543–555. 20623420 10.1080/09658211.2010.487051

