# Supplementary material for: False memories for true and false vaccination information form in line with pre‐existing vaccine opinions
Source: Appl Cogn Psychol. 2022 Oct 4:10.1002/acp.4002. Online ahead of print. doi: 10.1002/acp.4002 (PMC9537901; doi:10.1002/acp.4002)
Supplement: Supplementary file 1 — Appendix S1: Supporting Information. [file ACP-9999-0-s001.docx]

**Supplementary Materials**

**Examples of vignettes**


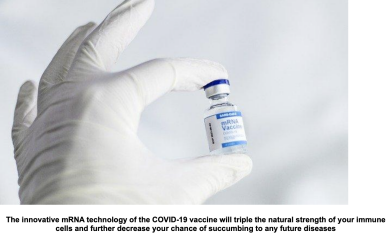


Figure A1 Example of pro-vaccine novel headline

**List of headlines in Study 1**

**Anti-vaccine novel headlines**

1. Episodes of ‘memory loss’ reported after receiving second COVID-19 vaccine dose increased this month.

2. The mRNA technology in the COVID-19 vaccine affects cell mutation and decreases your bone density.

3. The mRNA technology in the COVID-19 vaccine strains your immune cells, making you more susceptible to countless other illnesses.

4. Leaked: In order to maintain the illusion that the pandemic is under control, only 50% of COVID-19 vaccines being administered to the public actually contain the vaccine – the rest are simply placebos

5. COVID cover-up: Pfizer ex-employee who was “about to blow the whistle” on the unreported dangerous side effects of the COVID-19 vaccine was involved in a fatal crash. Police are treating the incident as suspicious.

**Pro-vaccine novel headlines**

1. The innovative mRNA technology of the COVID-19 vaccine will triple the natural strength of your immune cells and further decrease your chance of succumbing to any future diseases

2. The mRNA in the COVID-19 vaccine remains in your blood long enough to combat any other flu you may contract in the future

3. Reported ‘side-effects’ associated with the COVID jab are actually caused by vaccinerelated anxiety, and not the vaccines themselves – new study finds. The vaccines themselves do not cause any adverse side effects.

4. Regulators were so intent on providing a safe and effective COVID-19 vaccine that the vaccine trials consisted of six phases of testing rather than the usual three.

5. New study finds risk of lung cancer to be significantly reduced after two shots of COVID-19 vaccine

**Neutral true headlines**

1. Production for the new Batman movie to be released in 2022 was halted when its star, Robert Pattinson, tested positive for COVID-19

2. The Duke and Duchess of Sussex donated the earnings from the broadcast of their wedding to Feeding Britain U.K. to aid in COVID-19 relief, with a whopping donation of £90,000.

3. Tom Hanks was one of the first celebrities to contract COVID-19 back in March of 2020, and now encourages everyone to do their part in preventing the spread of the virus.

4. In the midst of the pandemic, New Zealand Prime Minister Jacinda Arden’s efforts against COVID-19 were rewarded when she won re-election.

5. After a two-day hospital visit following a positive COVID-19 test, President Donald Trump waved to supporters gathered outside, before heading back to the White House.

**List of headlines in Study 2**

**True anti-vaccine headlines**

1. AstraZeneca vaccine advice unlikely to change despite rate of rare clots 'doubling'.

2. Pfizer, Moderna vaccines show limited effectiveness against COVID-19 'Indian variant'.

3. Seychelles, world's most vaccinated nation, faces major COVID spike which suggests limited effectiveness of administered vaccines.

4. Reports of severe, life-threatening allergic reaction (anaphylaxis) occurring after Pfizer COVID-19 vaccine.

5. COVID vaccines associated with false-positive breast cancer result.

**True pro-vaccine headlines**

1. COVID-19: First nationwide data from Israel shows 95% protection from infection after two doses of Pfizer jab.

2. Pfizer-BioNtech booster vaccine significantly improves immune responses in patients with cancer.

3. Pfizer-BioNtech and AstraZeneca jabs effective against 'Indian variant' after two doses.

4. Vaccines may provide coronavirus immunity that lasts for years, finds study.

5. Benefits outweigh the risk: Risk of becoming seriously ill from COVID-19 much higher than risk of blood clots from COVID-19 vaccine.
